# Presentation of gastroesophageal junction adenocarcinoma with synchronous metastases at the small intestine. Could treatment with curative intent be considered? A case report

**DOI:** 10.1016/j.ijscr.2021.106164

**Published:** 2021-06-30

**Authors:** Biying Huang, Aristotelis Kechagias, Andrianos Tsekrekos, Andrea Lovece, Masaru Hayami, Ioannis Rouvelas

**Affiliations:** a Department of Upper Abdominal Surgery, Karolinska University Hospital, Stockholm, Sweden; b Department of Digestive Surgery, Kanta-Häme Central Hospital, Hämeenlinna 13530, Finland; c Division of Surgery, Department of Clinical Science, Intervention and Technology (CLINTEC), Karolinska Institutet, Stockholm, Sweden; d Division of General Surgery, IRCCS Policlinico San Donato, San Donato Milanese, Italy

**Keywords:** GI, Gastrointestinal, CT, Computed Tomography, FDG-PET, FluoroDeoxyGlucose - Positron Emission Tomography, GEJ, Gastroesophageal Junction, SCC, Squamous Cell Carcinoma, Esophageal cancer, Synchronous intestinal metastases, PET-CT, Curative treatment, Esophagectomy, Case report

## Abstract

**Introduction:**

Introduction of multimodality treatment as the standard of care for management of esophageal and gastroesophageal junction (GEJ) cancer over the last years has led to significant improvement in survival for patients with localized disease. Nevertheless, treatment with curative intent is not considered in the case of metastatic disease. We report a case of a locally advanced GEJ adenocarcinoma with solitary resectable synchronous metastases at the jejunum and a good response to neoadjuvant therapy followed by esophagectomy with curative intention.

**Case presentation:**

This is the case of a patient with poorly differentiated adenocarcinoma of the GEJ with synchronous metastases at the jejunum. The patient underwent extensive work-up including PET-CT. The metastases at the jejunum were completely resected during an initial staging laparoscopy and there was no evidence of further metastatic disease. The patient received chemotherapy and re-staging showed remarkable tumor response. Esophagectomy with curative intent was performed. Histopathology showed complete pathologic response after chemotherapy. Although our patient had a stage IV disease at presentation, he remained metastasis-free for a significant period of time, with no evidence of any distant recurrence during a follow-up of 16 months after esophagectomy.

**Discussion and conclusions:**

Synchronous metastasis to the small bowel from an esophageal carcinoma is a rare entity. Routine PET-CT in addition to conventional CT may assist in more precise staging of a patient with resectable disease. Stage IV esophageal cancer with limited and resectable metastatic disease and good tumor response to oncological therapy may be considered for treatment with potentially curative intent.

## Highlights

• Synchronous metastasis to the small bowel from an esophageal carcinoma is a rare entity.
• PET-CT in addition to conventional CT may assist in more precise staging of esophageal cancer.
• Esophageal cancer with resectable metastases may be considered for treatment with curative intent.

## 1 Introduction

The management of esophageal carcinoma has evolved significantly during the last years. The main innovation in the current treatment of resectable but locally advanced esophageal cancer is the use of neoadjuvant chemoradiation or peri-operative chemotherapy with the intent to increase the possibility of an oncologically complete resection and to improve disease free survival [1]. Nevertheless, curative intent treatment is not considered an option in the case of metastatic esophageal cancer [1]. The objective of this report is to present the first case, to our knowledge, of a locally advanced gastroesophageal junction (GEJ) adenocarcinoma with resectable synchronous metastases at the jejunum and a good clinical response to neoadjuvant therapy followed by esophagectomy with curative intention. Based on this case, we aim to discuss possible diagnostic and therapeutic pathways with the intent to achieve cure in selected patients with primarily resectable metastatic disease. This manuscript has been prepared according to the SCARE guidelines for surgical case reports [2].

## 2 Case presentation

**Fig. 1:**
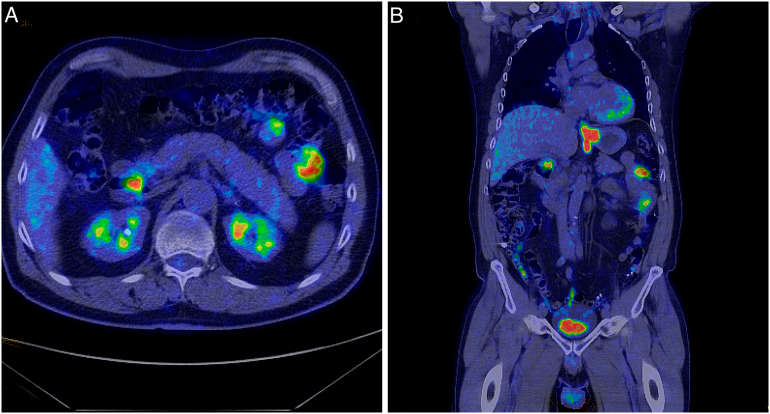
Initial PET-CT findings: high FDG-uptake in the primary gastroesophageal junction tumor as well as in three lesions in small bowel indicating metastatic disease. a. Axial section, b. Coronal section.

A 69-year-old male presented to his primary-care physician in March 2019 due to progressive dysphagia during a period of approximately one year. He was previously healthy, except for urolithiasis and benign prostatic hyperplasia. A barium swallow study was performed and showed a stricture at the distal esophagus. Further investigation with an upper-gastrointestinal (GI) endoscopy revealed a tumor at the distal esophagus and biopsies confirmed poorly differentiated adenocarcinoma. The patient was referred to our unit, Department of Upper Abdominal Surgery, Karolinska University Hospital, Stockholm, Sweden, which is a tertiary referral center for upper-GI malignancies. Subsequent computed tomography (CT) and fluorodeoxyglucose - positron emission tomography (FDG-PET) were performed. The case was thereafter discussed at our upper-GI multidisciplinary team meeting. The disease was classified as a 6 cm Siewert type-II tumor at the GEJ, with a clinical stage of T3-4a, N1 (due to a 15 mm lymph node alongside the lesser curvature of the stomach), and Mx due to equivocal lesions with elevated FDG-uptake at the ampulla of Vater and at two segments of the small intestine (Fig. 1). Further investigation with duodenoscopy did not confirm any lesion at the ampulla of Vater.

**Fig. 2:**
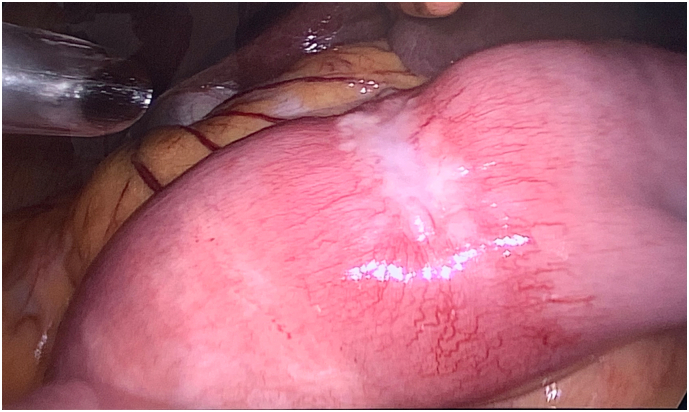
Finding in first diagnostic laparoscopy: suspicious lesions in jejunum.

**Fig. 3:**
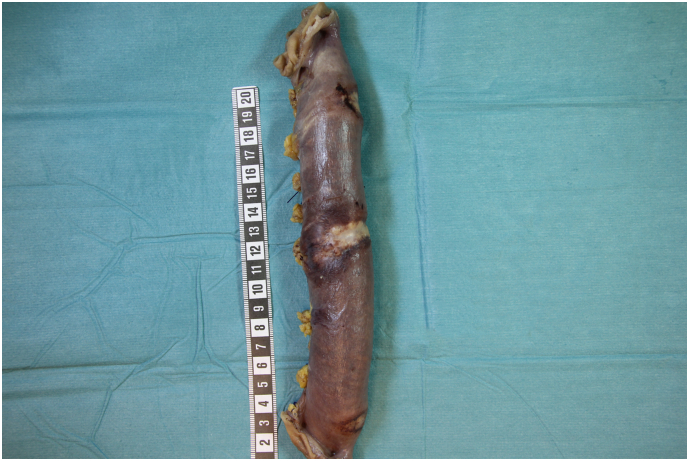
Specimen of resected segment of jejunum including all three suspicious lesions.

The patient underwent a diagnostic laparoscopy in April 2019, which revealed three suspicious lesions involving a 25 cm-long segment of the proximal jejunum (Fig. 2), starting approximately at 25 cm distally to the ligament of Treitz. There was no obvious, macroscopic, serosal invasion of the primary tumor, no signs of peritoneal carcinomatosis or ascites, and the duodenum was found to be normal. Because of risk for leakage by taking jejunal biopsies as well as to avoid future risk for bowel obstruction, the affected segment of the jejunum was resected through a mini laparotomy. The pathologic examination showed three separate, radically resected, poorly differentiated adenocarcinomas (3 × 1 cm, 5,5 × 1,5 cm and 3 × 1 cm, respectively, Fig. 3). Immunohistochemistry suggested that these tumors were most probably metastases of the primary GEJ tumor. The cytology from the abdominal washout taken during the laparoscopy was negative.

As the jejunal metastases were radically removed and there were no other indications of disseminated disease, neoadjuvant chemotherapy was decided with the intent for subsequent curative resection of the primary tumor. The patient received four cycles of Docetaxel, Oxaliplatin, Leucovorin and 5-flurouracil (FLOT). The patient tolerated the oncologic treatment well, except for some diarrhea (Grade 1 according to the common terminology for adverse events version 5). A new PET-CT after the completion of chemotherapy showed remarkable tumor response with only weak FDG-uptake in the primary tumor and the previously described regional lymph node. However, a new site of focal FDG-uptake appeared in another part of the small intestine at the left flank. Furthermore, there was also diffuse FDG-uptake in the left colon probably due to chemotherapy-induced colitis. The patient underwent another diagnostic laparoscopy, which did not show any suspicious findings. The anastomosis from the previous jejunum resection appeared as erythematous and fibrotic, corresponding to the suspicious lesion in the PET-CT.

**Fig. 4:**
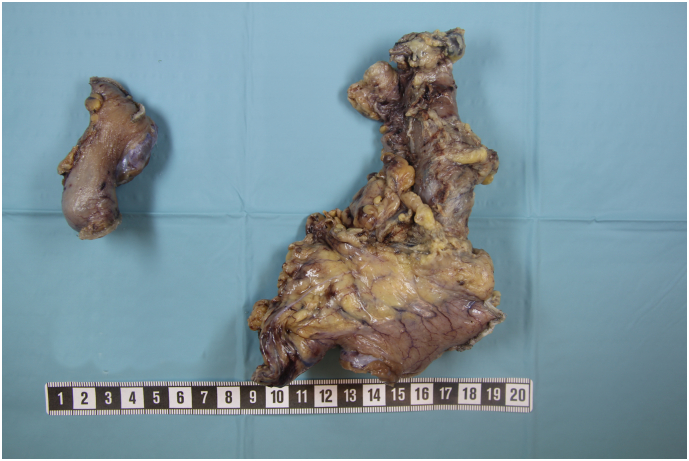
Specimen of resected primary tumor after esophagectomy inclusive redundant gastric conduit.

The patient subsequently underwent a minimally invasive Ivor Lewis esophagectomy with two-field lymphadenectomy and gastric tube interposition in August 2019. There were no complications intraoperatively. Fig. 4 shows the surgical specimen. An enhanced recovery program was implemented postoperatively and the patient was discharged on postoperative day 15. The postoperative course was uneventful, until the patient was re-admitted due to paralytic ileus on postoperative day 18, which was treated conservatively with gastrografin. The pathology examination showed complete tumor regression (ypT0N0 with 37 lymph nodes resected) and confirmed an R0 resection. The patient received adjuvant chemotherapy with additional four cycles of (FLOT). One year and four months after the esophagectomy there were no clinical or radiologic findings of tumor recurrence. At seventeen months post esophagectomy, he developed left-sided weakness and mild cognitive impairment. Magnetic resonance imaging revealed a 2,5 cm parietal tumor. There were no other signs of recurrence on CT-scan of the thorax and abdomen. The patient underwent surgical resection of the brain tumor in February 2021. Histopathology confirmed a radically resected metastasis of his previous GEJ cancer. He is now slowly recovering and he is awaiting complementary radiotherapy.

## 3 Discussion

Our case consisted of a patient with a poorly differentiated adenocarcinoma of the GEJ with synchronous metastases at the jejunum (stage IV disease). The patient underwent extensive work-up including a PET-CT, which is routine practice at our center for the last ten years. All the metastases at the jejunum were completely resected during the staging laparoscopy and there was no evidence of further metastatic disease. Therefore, we considered the possibility of treatment with curative intent. The patient received the same therapy as for patients presenting with locally advanced disease (stage III), which comprises neoadjuvant chemotherapy followed by re-staging and, in the case of stable disease, esophagectomy. Interestingly, there was a complete pathologic response to the neoadjuvant regimen despite the poor differentiation of the tumor's initial histology. Although our patient had a stage IV disease at presentation, he remained metastasis-free for a significant period of time, as there was no evidence of any distant recurrence during the initial 16 months after esophagectomy. The isolated brain recurrence, 17 months post esophagectomy, despite the complete pathologic response is most probably an expression of the given inadequate chemotherapy penetration across the blood-brain barrier [3].

Esophageal cancer with synchronous metastasis to the small bowel is a rare entity and there are very few data available in the literature. To the best of our knowledge all reported cases are summarized in a recent review by Ono et al. [4]. In total, there were 13 patients between 1988 and 2018 and all of them were male. Twelve had squamous cell carcinoma (SCC) and only one patient had an adenocarcinoma, yet, in this specific case the metastasis to small bowel was metachronous [5]. In one SCC case there were two metastatic lesions in the small bowel, while the rest had only one focus [4]. The most interesting finding was that almost all these patients (12 out of 13) presented due to an acute abdominal symptom caused by the intestinal metastases. Therefore, the presence of metastatic disease to the small bowel was revealed during the emergency treatment of a perforation (3 cases) or an obstruction (9 cases) [4]. Intestinal metastasis was an incidental finding only in one patient and it was noticed while placing the naso-jejunal feeding catheter during planned esophagectomy [6]. Bearing all the above in mind, one could summarize that the average profile of a patient with esophageal cancer and synchronous metastases to the small intestine includes an unplanned hospitalization due to acute etiology from the previously unknown metastases.

The diagnostic and treatment strategy that was used in our patient raises two issues. First, the importance of a routine PET-CT for the accurate staging of resectable, locally advanced esophageal and GEJ cancer, as an addition to conventional imaging should be advocated. Indeed, the management of patients with metastatic esophageal cancer (stage IV) differs as they commonly are not considered within the frame of potentially curable disease, unless included in relevant research protocols [1]. Palliative chemotherapy and radiotherapy or best supportive care (including insertion of esophageal stent), depending on patient's general condition, are the current options for synchronous distant metastatic disease, as curative esophagectomy is precluded [1]. In our center, an FDG-PET-CT-scan is routinely performed in order to more accurately stage locally resectable esophageal and GEJ cancer regardless the evidence or not of metastatic disease in the CT-scan. The rationale of routine PET-CT is to reduce the possibility of under-staging due to missed metastases in the conventional CT-scan. To our knowledge, our case is the only report in the literature where synchronous, solitary intestinal metastases originating from GEJ adenocarcinoma were detected during the staging work up and this is mainly attributable to the utilization of PET-CT. Unfortunately, the evidence in the literature concerning the additive role of PET-CT for resectable esophageal cancer in conventional radiology is still limited. A small series showed that PET-CT revealed distant metastases in 25% of the patients who had stage ≤III disease in the conventional CT-scans with the drawback of 7% of false-positive findings [7]. According to our experience with the herein presented case, we believe that further research is necessary in order to assess the potential benefit of PET-CT in the accurate staging of patients with resectable esophageal cancer.

A second issue to be considered is whether selected patients with stage IV disease should be offered therapy with potential curative intent. Our patient had resectable metastatic disease and once this was removed the management algorithm that was followed was the same as for a patient with primary stage III disease. The patient remained disease free for 16 months of meticulous follow-up, which is a considerable period of time for stage IV esophageal cancer at presentation. Our case could infer that treatment with curative intent could be an option for a patient with solitary distant metastases when there is a combination of resectable metastatic disease and good tumor response to the neoadjuvant regimen. It should be noted that current guidelines advocate chemotherapy-only for stage IV disease [1]. Nevertheless, we believe that curative management could be of consideration in well-selected and well-informed patients after thorough clinical staging and careful assessment of all the parameters, including patient performance status and co-morbidities, through a specialized multi-disciplinary evaluation.

## 4 Conclusion

Synchronous metastasis to the small bowel from an esophageal and GEJ carcinoma is a rare, but well-documented entity. A routine PET-CT in addition to conventional CT-scanning may assist in more accurate staging of a patient with locally resectable disease. Stage IV esophageal cancer with solitary resectable metastatic disease and good tumor response to oncological therapy may be considered for treatment with potentially curative intent.

## Sources of funding

This research did not receive any specific grant from funding agencies in the public, commercial, or not-for-profit sectors.

## Ethical approval

Not applicable.

## Consent for publication

Written informed consent was obtained from the patient for publication of this case report and accompanying images. A copy of the written consent is available for review by the Editor-in-Chief of this journal on request.

## Research registration

N/A.

## Guarantor

Ioannis Rouvelas

## Provenance and peer review

Not commissioned, externally peer-reviewed.

## CRediT authorship contribution statement

AL and IR conducted the literature search. BH, AK and IR drafted the manuscript. All authors contributed to the conception and design of the work. BH, AT, AL, MH and IR were involved in the management of the patient. All authors reviewed the manuscript and gave approval for publication of the final version.

## Declaration of competing interest

The authors declare that they have no competing interests.
